# Spatial Variations and Influencing Factors of River Networks in River Basins of China

**DOI:** 10.3390/ijerph182211910

**Published:** 2021-11-12

**Authors:** Yuanhuan Zheng, Chunxue Yu, Huabin Zhou, Jiannan Xiao

**Affiliations:** 1 Research Center for Eco-environmental Engineering, Dongguan University of Technology, Dongguan 523808, China; ZhengYH_dgut@163.com (Y.Z.); zhouhb010@163.com (H.Z.); 2 School of Environmental and Civil Engineering, Dongguan University of Technology, Dongguan 523808, China; mczx607@163.com

**Keywords:** river network characteristics, drainage density, water surface ratio, drainage accumulation threshold

## Abstract

Analysis of the spatial variations in river networks and the related influencing factors is crucial for the management and protection of basins. To gain insight into the spatial variations and influencing factors of river networks between large basins, in this study, three river basins from north to south in China (Songhua River Basin, Yellow River Basin and Pearl River Basin) were selected for investigation. First, based on a digital elevation model, different river networks with six drainage accumulation thresholds of three basins were extracted using ArcGIS. The optimal networks were determined through fitting the relationship between the accumulation threshold and related drainage density. Then, we used two indicators, drainage density and water surface ratio, to characterize the spatial variations of three basins. Finally, Pearson’s correlation coefficients were calculated between those two indicators and natural/human influencing factors. The results showed that drainage density and water surface ratio decreased from north to south in China and were negatively correlated with natural/human influencing factors. Drainage density was more influenced by natural factors than by human factors, while the opposite was true for water surface ratio. These findings may provide some basis for the management and protection of the river network.

## 1. Introduction

River networks play an essential role in shaping river morphology [1], maintaining biodiversity [2,3], and regulating storage and flood control [4,5]. Nowadays, river networks are threatened with a reduction in length and area due to both natural and human activities, leading to serious damage to their functions [6,7]. Therefore, the conservation and management of river networks has become the focus of current research [8,9].

Understanding river network characteristics is the basis for river network protection and management [10,11]. River network characteristics (e.g., drainage density, length, stream length, drainage texture etc.) are indicators that describe the river network in a basin, and many scholars use them to characterize the spatial patterns and variations of river networks [12,13,14,15]. Heasley et al. found that river network density can indicate differences in the density and spatial arrangement of network structures within each basin [16]. Kopp et al. assessed the spatial pattern of river networks using basin area, network length and drainage density [17]. The characteristics of river networks can be influenced by human and natural activities [18,19]. For example, Collins et al. analyzed the river network characteristics in the Colorado and found that increased rainfall under dry climatic conditions led to increase in vegetation, which suppressed sediment transport and decreased drainage density [20]. Shao et al. found that an increase the number of reservoirs in the Pearl River basin reduced the connectivity of the river network [21]. Lin et al. and Xu et al. found that, influenced by urbanization, the Yangtze River basin river networks in the downstream showed a local increase in overall surface ratio and drainage density, and an overall decrease [22,23]. However, most studies have focused on individual river networks, and few studies have analyzed spatial variation in river network characteristics across multiple river basins [24,25]. It is importance to analyze river network characteristics between multiple river basins. For example, the integrated management of large-scale rivers and lakes requires consideration of the differences between basins for comprehensive and effective management. The Yellow River, the second largest river in China, affects not only the Yellow River basin, but also the Haihe River basin and the Huaihe River basin. For inter-regional water transfer projects, the differences between basins need to be considered even more, as in the case of the South to North Water Transfer Project in China and the North to South Water Transfer Project in the USA. Therefore, there is a need to analyze the spatial variations and influencing factors of river networks in multiple river basins.

In this study, three river basins (the Songhua River Basin (SRB), the Yellow River Basin (YRB) and the Pearl River Basin (PRB)) across the north and south of China were selected as research basins. To obtain river network data, the river network was extracted using the ArcGIS Hydrological Analysis module based on digital elevation model (DEM) data. Considering that the river network extracted from the DEM would be influenced by the resolution of the image map and the algorithm [26], six drainage accumulation thresholds were designed to generate different river networks. The optimal threshold was selected to be determined through fitting the relationship between the drainage accumulation threshold and related drainage density. Two indicators, drainage density and water surface ratio, were used to analyze the spatial variations of river networks in different basins. Finally, the influence of natural activities (mean temperature and annual precipitation) and human activities (mean population density and gross domestic product (GDP)) were analyzed on the characteristics of the river networks. The present study may provide an understanding of the characteristics of China’s river network and its spatial variations in different basins.

## 2. Study Area and Methods

### 2.1. Study Area

**Figure 1:**
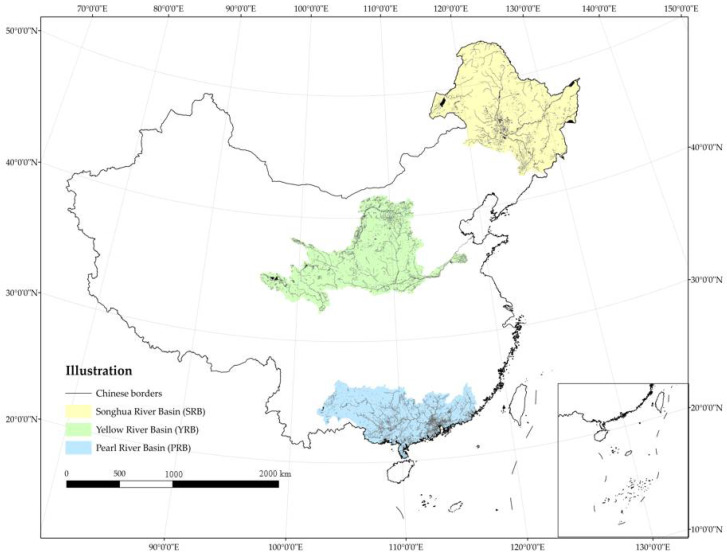
Location of the three basins and their river and lake distribution.

Three basins have been selected from the ten major water resource zones in China, north to south, based on different natural and social conditions. The distribution maps of rivers and lakes within the basins were extracted from the land use data, as shown in Figure 1. The basin zoning is based on the ten water resource zones in China. The yellow part indicates the SRB, the green part the YRB and the blue part the PRB. Three basins cover approximately 24% of China’s land area.

The SRB is located in northeast China, between 41°42′ and 51°38′ north latitude and 119°52′ and 132°31′ east longitude, covering four provinces. The SRB is surrounded by mountains on three sides, with the Changbai Mountains in the east, and the Daxinganling and Xiaoxinganling in the west and northeast, respectively. Also present are the low hills of the SRB and the basin of the Liao River basin in the south, and a hilly transition zone between the mountains and the plains. In the center is the Songnun Plain, an important agricultural area in China [27]. The SRB has a north-temperate monsoon climate with average temperatures of 2 °C. The annual precipitation in the basin is roughly 400~1000 mm, with a multi-year average of about 500 mm, decreasing from east to west [28].

The YRB is the geographical and ecological area affected by the Yellow River system between latitude 32°8′ and 41°50′ north and longitude 95°52′ and 118°3′ east, covering nine provinces [29]. The topography of the YRB is in a three-stage gradient, spanning from west to east across the Tibetan Plateau, the Inner Mongolian Plateau, the Loess Plateau and the predominantly Yellow Huaihai Plain. The western region has an average altitude of over 4000 m, with snow all year round and mainly glacial landforms; the central area is a loess landform with an altitude of between 1000–2000 m. The eastern region is dominated by the Yellow River alluvial plain [30]. The main climate of the YRB is semi-arid continental, with an average temperature of around 7 °C. Precipitation is concentrated in the south of the middle, upper and lower reaches, while inland areas receive little precipitation and evaporation is high [31].

The PRB is a composite basin consisting of four river systems: the Xijiang, Beijiang, Dongjiang and the Pearl River Delta rivers, between 18°10′ and 26°52′ north latitude and 102°15′ and 117°11′ east longitude. As a major river in the south, it covers six provinces as well as Hong Kong and Macau [32]. It is bounded by deltaic impact plains in the south-east, hills and basins in the center, the South China Sea in the south, the Yunnan-Guizhou Plateau in the west and the Nanling Mountains in the north. The climate in the basin is subtropical monsoonal with an average temperature of around 20 °C. There are high temperatures and high rainfall in summer and milder temperatures and low rainfall in winter, with an average annual rainfall of about 1200 to 2400 mm. Due to the high and concentrated rainfall, floods occur frequently in the middle and lower reaches [33,34].

### 2.2. Data Sources

This study used 90 m resolution DEM data from three basins in 2015, provided by the Institute of Geography, Chinese Academy of Sciences. Mean temperature and annual precipitation were chosen as natural indicators [5], and the data were obtained from the China Meteorological Data Network (http://data.cma.cn, accessed on 17 August 2021). Considering the fluctuation of meteorological data, the meteorological data of 2015, which is close to the average of a normal year, was chosen for this case. Mean population density and GDP were chosen as human activity indicators [23], with data from the Urban Statistics Yearbook. These four impact indicators were applied to carry out correlation analysis of river network characteristics in the study area. Details are shown in Table 1 below.

### 2.3. Methods

#### 2.3.1. Extraction of the River Network

There are proven techniques to extract river networks from DEM data [35,36,37,38]. The basic idea of DEM-based extraction of the river network is to carry out depression filling, flow direction calculations, flow statistics, determination of optimal drainage accumulation thresholds, and vectorization of the river networks.

(1) Depression filling: Original DEM image data can have some data errors resulting in depressions that interfere with the algorithm to produce unreasonable flow directions [39]. Therefore, before using ArcGIS for hydrological analysis, the depressions in the original DEM data should be filled in to obtain a depression-free DEM in preparation for the subsequent flow direction calculations.

(2) Flow direction calculations: One of the key steps in acquiring a river network is determining the direction of outflow for each element in the raster. Currently, a widely used algorithm for determining the flow direction is the D8 algorithm, which determines the flow direction by coding the eight neighboring grids of the central grid [40]. This method provides a simple and efficient simulation of river flow in its natural state.

(3) Flow statistics: The Flow Calculation module in ArcGIS enables the creation of a raster image of accumulated flows for each grid. The grid flow represents the accumulative flow from other grids into this grid. Areas of high flow raster indicate that surface runoff is more likely to form in that area.

(4) Determination of the optimal drainage accumulation threshold: The key to making the results of the extracted river network similar to the existing river system of the basin is to determine the optimal drainage accumulation threshold. In this paper, the optimal drainage accumulation threshold uses the point at which the relationship between the drainage accumulation threshold and the drainage density reaches a smoothing. Any grid above this threshold will be vectorization [41].

#### 2.3.2. River Network Characteristics Indicators

There are a number of indicators that describe the characteristics of a river network e.g., drainage density, stream order, stream length, bifurcation ratio, drainage frequency, drainage texture, form factor, circularity ratio, elongation ratio, etc. [25]. In this case, drainage density and water surface ratio are used to characterize the river network of a large basin.

Drainage density is the total length of the rivers within a unit area of a basin [14,42,43]. Drainage density reflects the density of the river systems in the basin. Drainage density is related to various factors, such as the climate, geological surface, vegetation cover, soil, hydrology, and human activities in the area. The formula is calculated as follows:

D_d_ = *L_R_*/*A* (1)

where D_d_ is drainage density and *L_R_* and *A* are the total length and total area of rivers in the basin, respectively.

Water surface ratio, an important indicator to describe the spatial characteristics of a basin, is the ratio of the area of rivers and lakes to the area of the basin. It is important to maintain an adequate water surface ratio for urban flood control and the ecosystem service function of water purification, climatic regulation and biodiversity conservation [44,45,46]. The water surface ratio (R) is defined as:

R = *A_w_*/*A_L_* (2)

where *A_w_* is the total area of rivers and lakes within a certain basin, and *A_L_* is the total size of the basin.

#### 2.3.3. Analysis of Influencing Factors

Pearson’s correlation coefficient (r) was used to analyze the correlation between river network characteristics and the four influencing indicators (mean temperature, annual precipitation, mean population density, and GDP). Pearson correlation analysis enables correlation analysis of linear relationships between variables [47,48]. The definition and calculation formula of r were, respectively:

(3) $$r=\frac{Cov(IF_{i},Area_{j})}{\left[S.D.(IF_{i})\times S.D.\left(Area_{j}\right)\right]}$$

(4) $$r=\frac{\sum \left(IF_{i}-\bar{IF_{i}}\right)\left(Area_{j}-\bar{Area_{j}}\right)}{\sqrt{\sum {\left(IF_{i}-\bar{IF_{i}}\right)}^{2}\sum {\left(Area_{j}-\bar{Area_{j}}\right)}^{2}}}$$

where $IF_{i}$ is the impact index of item *i*; $Area_{j}$ is the characteristic value of river network in the *jth* basin; *Cov* is the covariance function between the variables $IF_{i}$ and $Area_{j}$; *S.D.* is the standard deviation function; $\bar{IF_{i}}$ is the $IF_{i}$ variables of average; and $\bar{Area_{j}}$ is the $Area_{j}$ variables of the average.

## 3. Results and Discussion

### 3.1. Spatial Variations in Drainage Density

**Figure 2:**
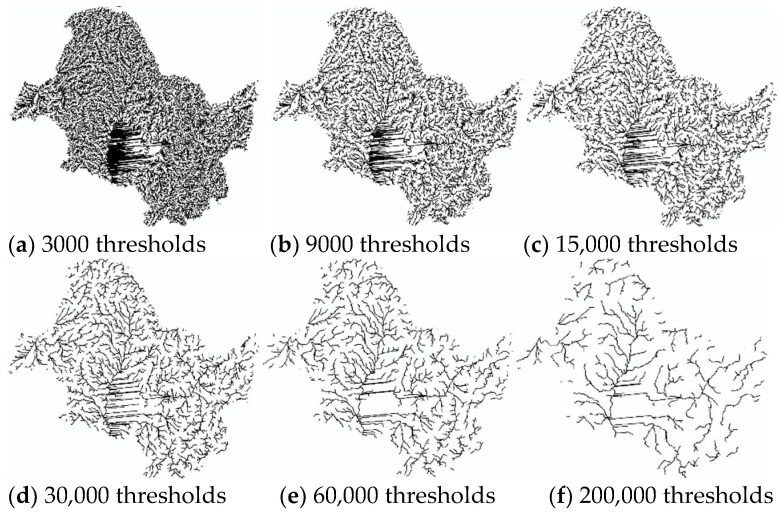
Map of the river network under different thresholds in the SRB. As the thresholds become larger, the slope network chain is gradually eliminated and the main rivers are revealed. The central plain with little elevation change generates a number of parallel river networks.

**Figure 3:**
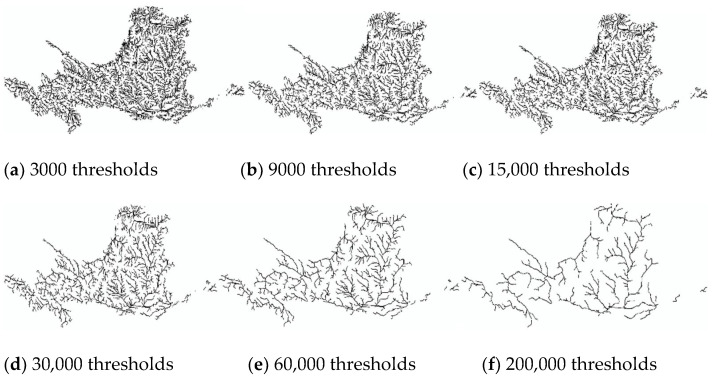
Map of river network under different thresholds of YRB. At thresholds between 3000 and 15,000 (**a**–**c**), the decrease in river network length is not visually significant. At thresholds above 30,000 (**d**–**f**), the river network changes significantly.

**Figure 4:**
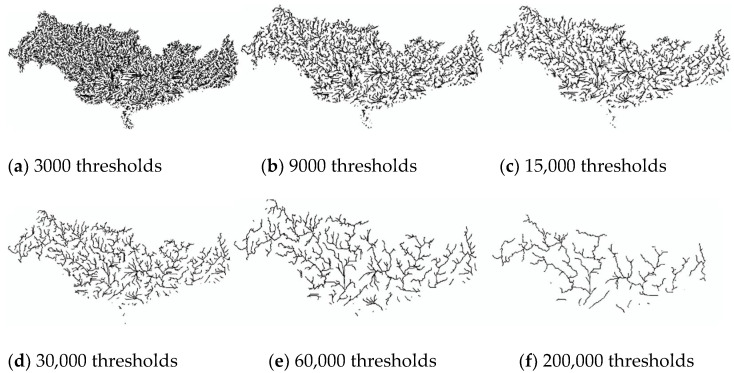
Map of the river network under different thresholds of PRB. Between 15,000 (**c**) and 200,000 (**f**), the river network varies significantly. The river network produced at larger thresholds (**d**–**f**) is sparser than that of the SRB and YRB.

Based on literature searches and practical studies, it can be found that there is a multiplicative power function relationship between drainage accumulation thresholds and drainage density [49,50]. This relationship enables us to establish a quantitative relationship between the threshold and the drainage density. In order to be able to visibly represent drainage density at different thresholds, six thresholds (3000, 9000, 15,000, 30,000, 60,000, 200,000) were chosen between 3000 and 200,000 thresholds (Figure 2 and Figure 3 and Figure 4). The drainage density for different thresholds for the length of the river network was calculated using Equation (1). The first order derivative of the power function relationship between the drainage accumulation threshold and the drainage density gives the rate of reduction in drainage density. Finally, combined with the river data in land use, an appropriate drainage density reduction rate was selected to determine the optimal drainage accumulation thresholds.

#### 3.1.1. Drainage Density in the SRB

Combining Figure 2 and Table 2, it can be found that the larger the threshold, the smaller the length of the extracted river network and the smaller the drainage density. After 30,000 thresholds, the reduction in density drainage is already below 0.001. By comparison, the images of the actual river data lie between the 60,000 and 200,000 threshold images. For this, we chose a threshold of −0.0001 for the drainage density reduction rate as the optimal drainage accumulation threshold. The optimal drainage accumulation threshold is 133,280 and the drainage density is 0.0250 km/km^2^.

**Figure 5:**
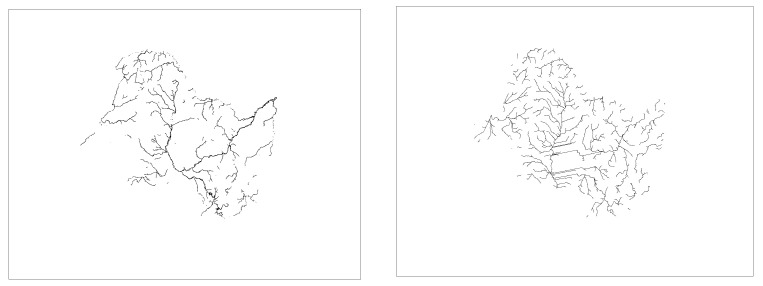
In the SRB, river in land use and the river under the optimal threshold. On the left are the rivers obtained from the land use data. On the right is the river network extracted with the threshold 133,280.

Comparing the two maps in Figure 5, it can be seen that the better simulation is shown in the northern Daxinganling region and the eastern Heilongjiang province region. The rivers in the Songhua River basin extracted from the optimal discharge accumulation threshold will be denser than those extracted from the land use data. There are three possible reasons for this difference: one is that the land use data extracted through remote sensing imagery is constrained by the resolution of imagery. Where the resolution is not high enough, the extracted river network will ignore small rivers. The second reason is that the plains are affected by human activity and the width and run-off of the rivers can be affected so that they are not extracted. The third reason is that a flaw in the algorithm produces unreasonable parallel river networks, some of which may exist.

#### 3.1.2. Drainage Density in the YRB

Analyze Table 3 and Figure 3, compare river data in land use, and select a threshold between 60,000 and 200,000. Considering the rate of drainage density reduction rate, a threshold of 126,590 is selected. Therefore, for the YRB, the optimal threshold is 126,590, with a river network length of 19,543.03 km, and the drainage density is 0.0246 km/km^2^.

**Figure 6:**
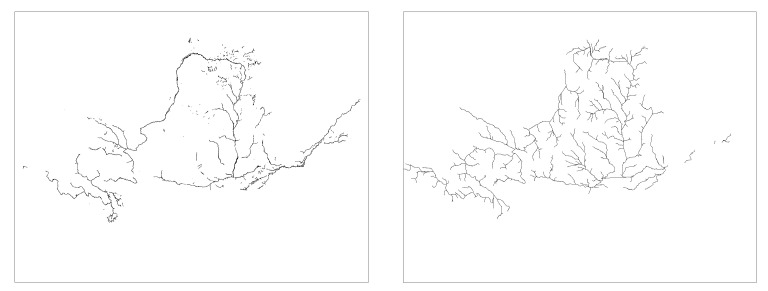
In the YRB, river in land use and the river under the optimal threshold. On the left are the rivers obtained from the land use data. On the right is the river network extracted with the threshold 126,590.

As can be seen in Figure 6, the distribution of the river network within the YRB is unevenly distributed, showing a sparse distribution in the west and a dense distribution in the east. The major tributaries of the Yellow River, the Fen, Jing, Wei and Huangshui rivers, are well modelled. In the middle and lower reaches of the Yellow River (Shanxi and Shaanxi Provinces), a similar river network can also be modelled from DEM data. In addition, topography exists where the riverbed is higher than the ground level on both banks in Bayannur in the Inner Mongolia Autonomous Region, Kaifeng in Henan Province and Shandong Province. This particular topography can seriously mislead the algorithm and lead to the river network extracted through the DEM being detached from the main stream.

#### 3.1.3. Drainage Density in the PRB

Analyze Table 4 and Figure 4 in the same way, compare the actual situation, which is close to the threshold between 60,000 and 200,000. The case where the drainage density reduction rate is 0.0001 as most optimal threshold is 115,740. Therefore, for the PRB, the optimal threshold is 115,740 with a river network length of 13,243.02 km, and the drainage density is 0.0228 km/km^2^_._

**Figure 7:**
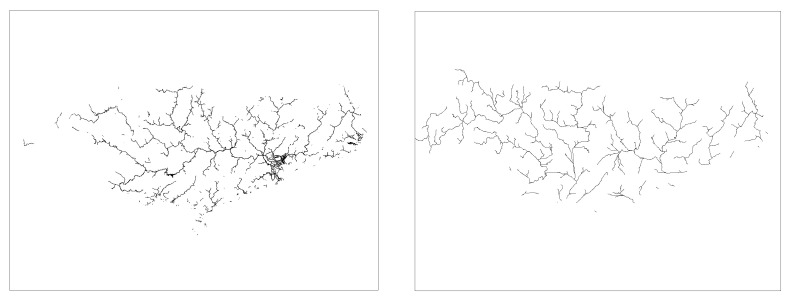
In the PRB, river in land use and the river under optimal threshold. On the left are the rivers obtained from the land use data. On the right is the river network extracted with the threshold 115,740.

By comparing the two maps in Figure 7, it can be seen that the PRB is well modelled for the Dongjiang and Beijiang rivers. For the source areas, the South and North Pan River are extracted better by DEM than from land use data. However, the Qian and Yu rivers, tributaries of the Xijiang, appear disconnected in Guiping and Guigang, Guangxi Province. By comparing the actual rivers with the topography and DEM data, there are two possible reasons for the disconnectedness of the simulation results. Firstly, the existing DEM resolution does not accurately describe the rivers generated by the complex topography, resulting in the disconnectedness of the extracted river network. The second is that the actual water system is too curved, due to severe human interference, to be represented in the simulation.

### 3.2. Spatial Variations in Water Surface Ratio

Data for rivers and lakes were calculated from land use data using ArcGIS. The total area of rivers and lakes was divided by the basin area using Equation (2) to obtain the water surface ratio. As can be seen in Table 5, there is little difference in river area between the three basins, but the difference in lake area and total area is more pronounced. The area of rivers and lakes in the SRB is approximately twice that of the YRB and three times that of the PRB. The water surface ratios of the SRB, YRB and PRB decrease in that order. The water surface ratio of the YRB is very close to that of the PRB, while that of the SRB is 1.5 times that of the YRB or PRB.

The SRB has a dense network of rivers and numerous lakes, with high water surface ratios indicating an abundance of water resources in the basin. To the left of the SRB basin, in Hulunbeier, Inner Mongolia, lies Hulun lake, the fifth largest lake in China, with an area of over 2000 km^2^. According to the 2010 survey results [51], the Heilongjiang province covered by the basin has 243 lakes larger than 1 km^2^ with a total area of 3241.3 km^2^; Jilin province has 181 lakes with a total area of 1402.8 km^2^. The central northeastern plain area is an important agricultural growing area with significant anthropogenic influence and a concentration of rivers and lakes.

The water surface ratio of the YRB is one third less than that of the SRB. The water resources of the YRB are mainly concentrated in the plateau area at the source of the Yellow River and in the Loop Plain area. The high plains of Qinghai Province are flat and slow flowing water and are well recharged by precipitation and ice melt water, creating an abundance of rivers and lakes. In the Loop region of Inner Mongolia and Ningxia, the rivers are long and curved, with well-developed tributaries that form numerous lakes.

The PRB has the lowest water surface ratio of the three main basins, but it is very close to that of the YBR. The PRB is generally rich in water resources, and the river network is relatively evenly distributed, but almost all the lakes are less than 1 km^2^. There are no lakes larger than 1 km^2^ in Guangxi province within the basin, and there is only one large lake of 5.5 km^2^ in Guangdong province [51]. The landscape of the PRB is predominantly hilly, with a high vegetation cover, and human activities are mainly concentrated in the PRB.

Overall, the SRB has a high drainage density and high water surface ratio. The YRB has a high drainage density and low water surface ratio, while the PRB has a low drainage density and low water surface ratio. Spatial differences in river networks exist in different basins and exist as a result of a combination of man-made and natural processes.

### 3.3. Correlation Analysis of River Network Characteristics and Influencing Factors

As shown in Table 6, drainage density and water surface ratio were negatively correlated with all four influencing factors. From a mathematical and statistical point of view, the higher the four influencing factors (annual precipitation, mean temperature, average population and GDP), the lower the drainage density and water surface ratio will be. The correlation coefficients between drainage density and these three (mean temperature, annual precipitation and mean population density) are close to −1. The correlation coefficient between water surface ratio and GDP also tended to be close to −1 and was highly significant.

The two influencing factors, annual precipitation and mean temperature, can be considered natural influences. The other two influencing factors, mean population density and GDP, can be regarded as human influences. Comparing the absolute values of the correlation coefficients shows that drainage density is slightly more influenced by nature than by humans. Conversely, the size of the water surface ratio is influenced more by humans.

Mean temperature and drainage density show a significant negative correlation with a correlation of −0.993. Mean temperature and water surface ratio are not statistically significant and correlate with a correlation coefficient of −0.768. The correlation coefficient between drainage density and annual precipitation was −0.978, which was statistically significant at a correlation level of 0.133. The correlation coefficient between water surface ratio and annual precipitation was −0.521 and it was significant at 0.651, indicating that they were not significantly correlated. Mean temperature affects vegetation cover, with high vegetation cover preventing soil erosion and increasing infiltration, thus reducing drainage density [20]. The low negative correlation between mean temperature/annual precipitation and water surface rate may indicate that natural influences contribute less positively than negatively to water surface rate. Changes in temperature affect rainfall as well as evapotranspiration, and various water-saving projects by people to increase the area of water storage will have a positive effect on the water surface rate.

The negative correlation between average population density and drainage density is also high at −0.986 and is correlated at the 0.108 level. The correlation coefficient between average population density and water surface ratio is −0.800, which is correlated at the 0.410 level. Watersheds with high levels of anthropogenic activity modify the river to a high degree; the width of the river shrinks, tributaries increase, and runoff becomes smaller, thus not being extracted. The correlation coefficient between drainage density and GDP is −0.850, which is relevant at the 0.353 level. The correlation coefficient between surface area and GDP is −0.967, which is significant at the 0.165 level, and areas with a developed GDP take up more of the former rivers and lakes, which can have a negative impact on drainage density and surface area. The construction of reservoirs and hydraulic facilities also changes the state of incoming water and affects the state of the rivers and lakes downstream.

## 4. Conclusions

There are a number of limitations and directions for future improvement on the work presented in this paper. For the river network extraction, it is influenced by two factors: one is the resolution of the DEM and the other is the drainage accumulation threshold [26]. This paper focuses on the drainage accumulation thresholds for river network extraction. However, the resolution of DEM can indirectly affect the accuracy of river network extraction by influencing the drainage accumulation threshold. If higher resolution data are available, a rational delineation of large basins and the use of multiple thresholds for river network extraction could be explored in the next step of the work. For river network analysis, the uncertainty of influences such as climate change, urbanization development and other influencing factors does not allow for dynamic analysis of changes in river network characteristics based on the natural/human activities of a particular time [52,53,54]. In the next step of work, analysis of river network characteristics under uncertainty scenarios such as climate change can be considered.

The aim of this paper is to investigate the spatial differences in river networks of the three river basins in China and the factors that influence them. The findings show a general downward trend in drainage density as well as water surface ratio from north to south in Chinese basins. River network characteristics are significantly influenced by temperature, rainfall, population density and GDP, and these effects are all negatively correlated. Overall, comparing the differences in environmental factors in the spatial characteristics of river networks across multiple basins can provide some guidance when it comes to the conservation of river networks in multiple basins.

## Figures and Tables

**Table 1:** Values of the four indicators in the three basins in 2015.

Basin	Mean Temperature (°C)	Annual Precipitation (mm)	Mean Population Density (Person/km^2^)	GDP (Trillion RMB)
SRB	2.24	479.68	63.88	5.25
YRB	7.34	398.41	147.15	8.85
PRB	20.46	1770.95	318.61	10.67

Note: SRB represents the Songhua River Basin; YRB represents the Yellow River Basin; PRB represents the Pearl River Basin.

**Table 2:** Relationship between drainage density and drainage accumulation thresholds in the SRB.

Total Basin Area (km^2^)	Drainage Accumulation Threshold (10^3^ Threshold)	Length of River Network (km)	Drainage Density (km/km^2^)	Dd Reduction Rate
934,802	3	195,291.46	0.2089	−0.03553
	9	96,828.45	0.1036	−0.00649
	15	72,597.59	0.0777	−0.00294
	30	49,069.21	0.0525	−0.00101
	60	34,208.96	0.0366	−0.00034
	133.28	23,336.27	0.0250	−0.00010
	200	19,408.19	0.0208	−0.00005

**Table 3:** Relationship between drainage density and drainage accumulation thresholds in the YRB.

Total Basin Area (km^2^)	Drainage Accumulation Threshold (10^3^ Threshold)	Length of River Network (km)	Drainage Density (km/km^2^)	Dd Reduction Rate
795,044	3	135,871.10	0.1709	−0.02899
	9	76,437.66	0.0961	−0.00549
	15	58,298.67	0.0733	−0.00253
	30	40,478.66	0.0509	−0.00089
	60	28,261.68	0.0355	−0.00031
	126.59	19,543.03	0.0246	−0.00010
	200	15,734.78	0.0198	−0.00005

**Table 4:** Relationship between drainage density and drainage accumulation thresholds in the PRB.

Total Basin Area (km^2^)	Drainage Accumulation Threshold (10^3^ Threshold)	Length of River Network (km)	Drainage Density (km/km^2^)	Dd Reduction Rate
578,960	3	84,825.06	0.1465	−0.02449
	9	48,316.58	0.0835	−0.00468
	15	37,073.07	0.0640	−0.00217
	30	26,033.59	0.0450	−0.00076
	60	18,310.22	0.0316	−0.00027
	115.74	13,243.02	0.0228	−0.00010
	200	10,171.17	0.0176	−0.00004

**Table 5:** River and lake areas and water surface ratios in the three major basins in 2015.

Basin	River Area (km^2^)	Lake Area (km^2^)	Total Area of Rivers and Lakes (km^2^)	Water Surface Ratio
SRB	4032.81	7387.25	11,420.06	0.01221
YRB	3829.82	2491.86	6321.68	0.00795
PRB	3926.60	448.43	4375.03	0.00755

**Table 6:** Pearson correlation coefficients of drainage density with water surface ratio and four influencing factors.

		Mean Temperature (°C)	Annual Precipitation (mm)	Mean Population Density	GDP (Trillion Yuan)
Drainage density	Pearson related	−0.993	−0.978	−0.986	−0.850
	Sig.	0.075	0.133	0.108	0.353
Water surface ratio	Pearson related	−0.768	−0.521	−0.800	−0.967
	Sig.	0.442	0.651	0.410	0.165

## Data Availability

Data sharing not applicable.
